# Intraoperative Ultrasound: Emerging Technology and Novel Applications in Brain Tumor Surgery

**DOI:** 10.3389/fonc.2022.818446

**Published:** 2022-02-01

**Authors:** Giuseppe Roberto Giammalva, Gianluca Ferini, Sofia Musso, Giuseppe Salvaggio, Maria Angela Pino, Rosa Maria Gerardi, Lara Brunasso, Roberta Costanzo, Federica Paolini, Rina Di Bonaventura, Giuseppe Emmanuele Umana, Francesca Graziano, Paolo Palmisciano, Gianluca Scalia, Silvana Tumbiolo, Massimo Midiri, Domenico Gerardo Iacopino, Rosario Maugeri

**Affiliations:** ^1^ Neurosurgical Clinic, AOUP “Paolo Giaccone”, Post Graduate Residency Program in Neurologic Surgery, Department of Biomedicine Neurosciences and Advanced Diagnostics, School of Medicine, University of Palermo, Palermo, Italy; ^2^ Department of Radiation Oncology, REM Radioterapia srl, Catania, Italy; ^3^ Section of Radiology, Department of Biomedicine Neurosciences and Advanced Diagnostics, School of Medicine, University of Palermo, Palermo, Italy; ^4^ Department of Neurosurgery, Fondazione Policlinico Universitario "A. Gemelli" IRCCS, Università Cattolica del Sacro Cuore, Rome, Italy; ^5^ Department of Neurosurgery, Cannizzaro Hospital, Trauma Center, Gamma Knife Center, Catania, Italy; ^6^ Department of Neurosurgery Highly Specialized Hospital and of National Importance “Garibaldi”, Catania, Italy; ^7^ Division of Neurosurgery, Villa Sofia Hospital, Palermo, Italy

**Keywords:** ioUS = intraoperative ultrasound, CEUS (contrast-enhanced ultrasound), brain tumor surgery, intraoperative ultrasound, neuronavigation

## Abstract

Intraoperative ultrasound (IOUS) is becoming progressively more common during brain tumor surgery. We present data from our case series of brain tumor surgery performed with the aid of IOUS in order to identify IOUS advantages and crucial aspects that may improve the management of neurosurgical procedures for brain tumors. From January 2021 to September 2021, 17 patients with different brain tumors underwent brain tumor surgery aided by the use of IOUS. During surgery, the procedure was supported by the use of multiples ultrasonographic modalities in addition to standard B-mode: Doppler, color Doppler, elastosonography, and contrast-enhanced intraoperative ultrasound (CEUS). In selected cases, the use of IOUS during surgical procedure was combined with neuronavigation and the use of intraoperative fluorescence by the use of 5-aminolevulinic acid (5-ALA). In one patient, a preoperative ultrasound evaluation was performed through a former iatrogenic skull defect. This study confirms the role of IOUS in maximizing the EOR, which is strictly associated with postoperative outcome, overall survival (OS), and patient’s quality of life (QoL). The combination of ultrasound advanced techniques such as Doppler, color Doppler, elastosonography, and contrast-enhanced intraoperative ultrasound (CEUS) is crucial to improve surgical effectiveness and patient’s safety while expanding surgeon’s view.

## Introduction

Ultrasound (US) is an imaging technique widely used in medicine, thanks to the variety of information that it can provide together with its simplicity of use. The use of US in neurosurgery is strongly limited by the interposition of the skull; thus, the use of US in the adult is limited to transcranial Doppler for the evaluation of intracranial blood flow. Conversely, brain surgery offers a unique opportunity to employ intraoperative ultrasound (IOUS); in fact, the acoustic windows offered by craniotomy let US penetrate the brain, thus directly visualizing neural structures and supporting the surgical procedure (1).

The first application field of IOUS is represented by the neuro-oncological surgery. Since the 1980s, several studies have shown that IOUS is capable of accurately identifying brain tumor during neurosurgical procedures and facilitating tumor resection, both in case of intra- and extra-axial lesions. Since its development, IOUS allows surgeons to maximize the extent of resection while preserving normal brain parenchyma and, as a consequence, neurological functions, reducing the mortality and increasing the progression-free survival (PFS) (2–9).

B-mode is the simplest modality of US imaging; it is a 2-D imaging used to identify the lesions and their relationships with normal brain parenchyma and vascular structures. It is largely used as the first technique during IOUS, and then, it can be integrated with other advanced US techniques in order to realize a multimodal approach, with both anatomical and functional imaging (10).

As regard advanced US techniques, anatomical information provided by standard B-mode US may be extended by the use of different other techniques. In particular, Doppler IOUS differs from standard B-mode, since it does not provide an anatomical imaging but rather functional information about blood flow. The visualization of Doppler IOUS can be enhanced by the color techniques (color Doppler and power Doppler) in order to enrich B-Mode anatomical images with functional information about vascularization (11).

In relation to vascularization, intraoperative contrast-enhanced ultrasound (CEUS) is an advanced technique that adopts an intravenous ultrasound contrast agent made of gas-filled microbubbles in order to provide functional information about tumor biological characteristics through a direct visualization of its pattern of vascularization (11–15).

As regards the tactile differentiation of tumoral tissue from normal brain parenchyma, elastosonography is an advanced US technique that provides information about tissue stiffness through the evaluation of returned ultrasound echoes before and after a specific compression (5, 16).

Since the real-time images and the lack of conventional anatomical planes, IOUS visualization may be hostile and tricky for the untrained neurosurgeon. Given the availability of neuronavigation systems, in recent years, navigated IOUS has been developed in order to match IOUS imaging with neuronavigation. Navigated IOUS allows the fusion of preoperative MRI with IOUS imaging through the navigation of US probes. In this way, it is possible for the neurosurgeon to visualize IOUS imaging as overlay on brain MRI during real-time neuronavigation, thus improving the reliability of IOUS and the visualization of EOR against preoperative imaging (11, 17–22).

In the last years, neurosurgeons again performed US, thanks to the development of the aforementioned techniques. Moreover, evidence regarding the benefits of IOUS is strong; however, it still represents an uncommon technique, which is only used in a small number of centers. Through the evaluation of the aforementioned parameters, IOUS may influence surgical strategy and, consequently, the postoperative outcome, thanks to a multimodal approach for image guidance in brain tumor surgery. The purpose of this study was to analyze the characteristics of IOUS through data collected during our experience in order to evaluate the impact of IOUS in brain tumor surgery and its crucial aspect for a better intraoperative management of brain tumor patients.

## Materials and Methods

### Patient Population and Study Criteria

This is a retrospective case series of 17 consecutive patients who underwent brain surgery aided by IOUS for different brain tumors at our institution from January 2021 to September 2021. All patients underwent IOUS during surgical resection of different intracranial lesions: six patients suffered from meningioma, five from glioblastoma, one from breast cancer cerebellar metastasis, two from lung cancer metastases, one from colon cancer metastasis, one from embryonal tumor (medulloblastoma), and one from cerebellar diffuse large B-cell lymphoma. Moreover, one of these patients who was affected by right extra-axial lesion underwent ultrasound evaluation also before surgery, due to a previous iatrogenic skull defect.

The patients’ eligibility criteria were the following: the presence of brain lesion, the surgical indications for craniotomy, a craniotomy large enough for the probe placement, patient’s given informed consent, and the presence of a good acoustic window for ultrasound acquisition after craniotomy. Patients who did not meet such eligibility criteria were excluded.

The indications to the use of intraoperative ultrasound were the following: preoperative evaluation, anatomical study of tumoral lesion, evaluation of the relationships between tumor and brain parenchyma, study of the tumor stiffness, study of the tumor vascularization, study of brain vessels, and study of the residual tumor volume.

### Study Equipment

A latest generation ultrasound device (Esaote MyLab Twice, Italy) with a 3–11 MHz linear probe (Esaote LA332—Genova, Italy) was used, and multiple ultrasonographic modalities were adopted: these were B-mode, color Doppler Doppler, elastosonography, and contrast-enhanced intraoperative ultrasound (CEUS). CEUS was performed through the administration of ultrasound contrast agent (UCA) made with sulfur hexafluoride-filled microbubbles (SonoVue—Bracco Imaging, Italy). In selected cases, IOUS was combined with neuronavigation (Medtronic StealthStation S8—Minneapolis, USA) and the administration of 5-aminolevulinic acid (5-ALA) as tumor-specific fluorescent dye.

## Results

In total, 17 patients (7 men and 10 women) were included, and their data were retrospectively collected. Patients’ demographics and characteristics are summarized in **Table 1**. Mean age was 63.6 ± 11.1 years. All patients had been newly diagnosed with brain tumor on preoperative imaging. Among them, six patients (35,3%) were diagnosed with meningioma, three of which were fibrous meningiomas (grade I, WHO 2016), two meningothelial meningiomas (grade I, WHO 2016) (one of them presenting transposition areas to atypical meningioma), and one transitional meningioma; five patients (29.4%) were diagnosed with glioblastoma (grade IV, WHO 2016); one patient (5.9%) was diagnosed with breast cancer metastasis; two patients (11.8%) were diagnosed with lung cancer metastasis; one patient (5.9%) was diagnosed with colon cancer metastasis; one patient (5.9%) was diagnosed with medulloblastoma (grade IV, WHO 2016); and one patient (5.9%) was diagnosed with cerebellar diffuse large B-cell lymphoma.

**Table 1:** Demographic data of enrolled patients.

Case No	Age, Sex	Localization	Pathological diagnosis	Time	Ultrasound techniques						EOR	Purposes and advantages
					Doppler US	Color-Doppler US	CEUS	Elasto	Neuronav.	5-ALA		
***1***	56, F	Fronto-temporal, left	Fibrous meningioma	Intra-op	x	x	x	x			GTR	Craniotomy exposure, identification of the lesion, anatomical landmarks and vital structures, evaluation of brain shift, brain deformation, complete resection, post-resection cerebral blood-flow, and vasospasm evaluation
***2***	70, F	Frontal, left	Meningothelial meningioma	Intra-op	x	x	x	x			GTR	Craniotomy exposure, identification of the lesion, anatomical landmarks and vital structures, evaluation of brain shift, and brain deformation
***3***	81, F	Parietal, right	Glioblastoma	Intra-op	x	x	x	x		x	GTR	Craniotomy exposure, identification of the lesion, anatomical landmarks and vital structures, evaluation of brain shift and brain deformation, enlargement of the microsurgical resection, evaluation of EOR
***4***	74, M	Fronto-temporo-parietal, right	Glioblastoma	Intra-op	x	x	x	x		x	GTR	Craniotomy exposure, identification of the lesion, anatomical landmarks and vital structures, evaluation of brain shift, brain deformation, microvasculature and “en passant” brain vessels, enlargement of the resection, evaluation of EOR
***5***	50, M	Temporo-parietal, right	Fibrous meningioma	Pre-op	x	x	x	x				Identification of the lesion, study of vascular supply, anatomical landmarks and vital structures, surgical planning
				Intra-op	x	x	x				GTR	Craniotomy exposure, identification of the lesion, anatomical landmarks and vital structures, evaluation of brain shift, brain deformation, complete resection, avoidance of vascular lesions, evaluation of post-resection cerebral blood-flow and vasospasm
***6***	73, M	Fronto-temporo-parietal, right	Glioblastoma	Intra-op	x	x	x		x	x	GTR	Craniotomy exposure, identification of the lesion, anatomical landmarks and vital structures, evaluation of brain shift and brain deformation, enlargement of the microsurgical resection, evaluation of EOR
***7***	73, M	Parietal, right	Meningothelial meningioma	Intra-op	x	x	x				GTR	Craniotomy exposure, identification of the lesion, anatomical landmarks and vital structures, evaluation of brain shift, brain deformation, complete resection, evaluation of post-resection cerebral blood flow and vasospasm, post-resection cerebral blood flow and vasospasm evaluation
***8***	53, F	Fronto-temporal, right	Transitional meningioma	Intra-op	x	x	x	x	x		GTR	Craniotomy exposure, anatomical landmarks and vital structures, evaluation of total resection, post-resection cerebral blood flow and vasospasm evaluation
***9***	68, M	Temporal, right	Glioblastoma	Intra-op	x	x	x	x	x		STR	Craniotomy exposure, identification of the lesion, anatomical landmarks and vital structures, evaluation of brain shift and brain deformation, enlargement of the microsurgical resection, evaluation of EOR
***10***	60, F	Cerebellar, left	Breast cancer cerebellar metastasis	Intra-op	x	x	x				GTR	Craniotomy exposure, identification of the lesion, anatomical landmarks and vital structures, planning of the surgical corridor, evaluation of EOR
***11***	48, M	Cerebellar, left	Medulloblastoma	Intra-op	x	x	x				STR	Craniotomy exposure, identification of the lesion, anatomical landmarks and vital structures, planning of the surgical corridor, evaluation of residual tumor volume
***12***	40, F	Parietal, left	Glioblastoma	Intra-op	x	x	x		x	x	GTR	Craniotomy exposure, identification of the lesion, anatomical landmarks and vital structures, evaluation of microvasculature, evaluation of brain shift and brain deformation, enlargement of the microsurgical resection, evaluation of EOR
***13***	60, M	Cerebellopontine angle, right	Lung cancer cerebellar metastasis, whit cerebellar abscess	Intra-op	x	x	x				GTR	Craniotomy exposure, identification of the lesion, differentiation of cerebellar abscess, anatomical landmarks and vital structures, evaluation of EOR
***14***	72, F	Parietal, left	Lung cancer metastasis	Intra-op	x	x	x				GTR	Craniotomy exposure, identification of the lesion, anatomical landmarks and vital structures, evaluation of brain shift and brain deformation, evaluation of EOR
***15***	72, F	Parietal, left	Fibrous meningioma	Intra-op	x	x	x	x			GTR	Craniotomy exposure, identification of the lesion, anatomical landmarks and vital structures, evaluation of total resection
***16***	74, F	Cerebellopontine angle, left	Diffuse large B-cell lymphoma	Intra-op	x	x	x				GTR	Craniotomy exposure, identification of the lesion, anatomical landmarks and vital structures, avoidance of vascular lesions, planning of the surgical corridor, evaluation of total resection
***17***	58, F	Parietal, right	Colon cancer metastasis	Intra-op	x	x	x				GTR	Craniotomy exposure, identification of the lesion, anatomical landmarks and vital structures, evaluation of brain shift and brain deformation, evaluation of EOR

Elasto, elastonography; Neuronav., neuronavigation; EOR, extent of resection; GTR, gross total resection; STR, subtotal resection.

All patients underwent standardized protocol of intraoperative ultrasound with B-Mode US, Doppler, color Doppler ultrasonography, and CEUS; strain elastosonography was performed in eight patients (47.1%). 5-ALA was administered as tumor-specific fluorescent dye in all patients diagnosed with glioblastoma (29.4%). In four cases (23,5%), IOUS was matched with neuronavigation according to a coplanar indirect-navigated technique previously described (23); among them, three (75%) suffered from glioblastoma and one (25%) from transitional meningioma. In one patient (5.9%), a preoperative IOUS evaluation was performed through an acoustic window given by a previous iatrogenic skull defect in order to anticipate the following surgical strategy. Gross total resection was obtained in 15 cases (88.2%); subtotal resection (STR) was obtained in two cases (11.8%) due to a premature interruption of tumor resection because of the proximity to brain eloquent areas and the reduction in intraoperative motor evoked potential.

### Postoperative Outcome

Among the five patients with glioblastoma, three showed a good postoperative outcome without complications; two patients developed neurological impairments after surgery. One of them presented with altered consciousness, left upper limb monoplegia, and left lower limb paresis; he was then transferred to another institution for palliative care. The other one died after a multiorgan failure during postoperative care in the Intensive Care Unit. As regard six patients diagnosed with meningioma, three of them (50%) showed a good postoperative outcome without complications. Two patients developed neurological impairments after surgery: one of them developed a temporary palsy of the cranial nerve VII, and the other one developed right hemiparesis and motor aphasia. The only patient with medulloblastoma developed an incomplete palsy of the right cranial nerves VI and VII after surgery. The other patients showed a good postoperative outcome and were discharged without complications.

## Discussion

Intraoperative imaging techniques play a central role during neurosurgery procedures; among them, IOUS is becoming more and more adopted because it provides real-time direct visualization of brain tumors. Brain parenchyma has specific viscoelastic features, which allows exceptional US propagation, also because US beam is not attenuated by other interposed tissues (24).

IOUS gives the neurosurgeon an opportunity that other intraoperative imaging techniques do not. Since it can be repeated several times during surgery, IOUS can guide step-by-step the surgical procedure and it can overcome the errors produced by the phenomena of “brain shift” and “brain deformation”, from which standard neuronavigation suffers (2, 3, 25–28). Moreover, IOUS is a cost-effective, quick, and easy-to-use technique and can be combined with other advanced US modalities and intraoperative technique (1–4, 6, 7, 12, 13, 23, 29, 30).

In our preliminary experience, IOUS demonstrated its effectiveness during brain surgery, since it improved the identification of tumor localization and the differentiation of tumor from normal brain parenchyma, and of the direct visualization of peritumoral microvascularization. In this way, the information given by IOUS better defined the surgical strategy and precisely guided the tumor removal, maximizing the EOR and improving patient’s safety. Moreover, IOUS allowed the direct evaluation of tumor remnants, thus reducing the residual tumor volume (RTV) after surgery. EOR and RTV are considered as major independent predictors of overall survival (OS); since GTR is considered the goal to achieve in the treatment of brain tumor and it is directly related to OS, every possible effort has to be pursued in order to safely obtain the maximal extent of resection. However, a surgeon’s perspective may be inaccurate to intraoperatively evaluate the RTV during tumor removal: in this case, IOUS and CEUS are capable of giving an immediate confirmation of the real EOR and, thus, the RTV (2–4, 25, 26, 31). In our experience, GTR was achieved in most cases; this was made possible, as IOUS was employed in multiple phases of surgical procedure: after bone flap removal but before opening the dura mater, IOUS evaluated whether the target lesion was centered; after opening of the dura mater, ultrasound helped to identify the lesion, important anatomical landmarks, and its vascularization. During surgery, ultrasound was used from time to time to adapt surgery to the induced changes in the brain. At the end of surgery, IOUS was crucial to identify any tumor remnants and to confirm the integrity of vascular structures, and potential brain damage.

In our service, we routinely use the IOUS guidance for brain tumor resection, in accordance with the abovementioned inclusion criteria. In the operating room, IOUS is performed by neurosurgeons with the cooperation of neuro-radiologist for the interpretation of images, real-time feedback about IOUS setup, and potential artifacts avoidance.

In fact, US could give raise to multiple artifacts, which may interfere with the correct interpretation of the images. For this reason, some technical expedients may guarantee a higher quality of IOUS imaging. According to our experience, a proper preoperative arrangement of the probe inside the sterile drape is mandatory, avoiding the creation of air bubble that could limit the correct visualization of IOUS. During the intraoperative placement of the probe, an accurate irrigation of the surgical field ensures a correct acoustic coupling and removes remnants of artifactual materials. In particular, hemostatics, gel foams, and cottonoids should be limited and used only after IOUS, since they could severely artifact IOUS image.

**Figure 1:**
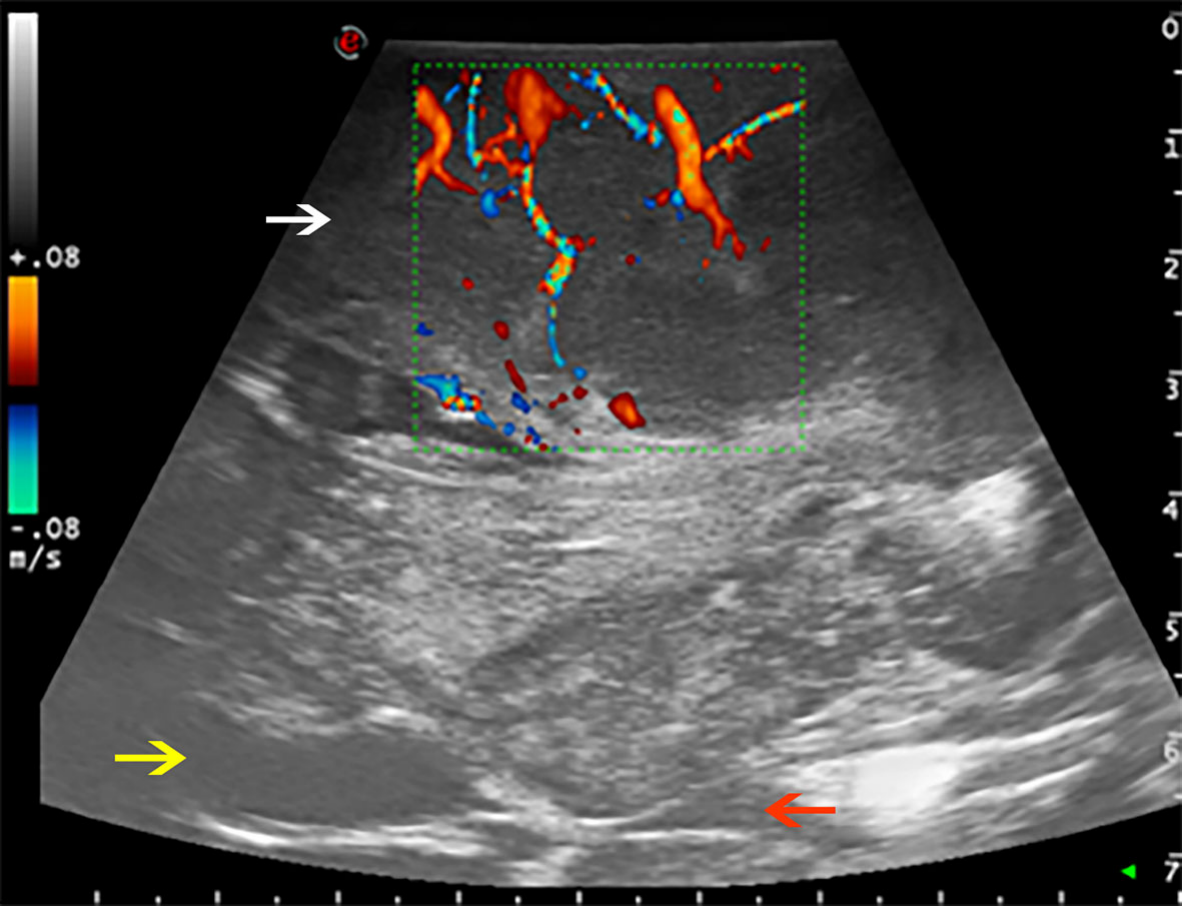
Color Doppler used to evaluate the perfusion pattern of a fronto-temporo-parietal GBM (case no. 6). White arrow: glioblastoma. Yellow arrow: frontal horn of lateral ventricle. Red arrow: third ventricle.

As regard US techniques, B-mode was employed as the first ultrasonographic tool for the morphological study of brain lesions, which has been further investigated by Prada et al. (13). According to their findings, in patients suffering from GBM, the lesion appeared hyperechogenic and characterized by the coexistence of different areas of necrosis, cysts, bleedings, and irregular dense tissue. Instead, meningiomas were hyperechogenic compared to the normal brain tissue, with a homogeneous pattern; tumor margins were always clearly detectable. The perfusion pattern and the vascular supply were analyzed thanks to other techniques, such as the Doppler and color Doppler ultrasonography. These techniques are capable of immediately identifying arterial supply to the tumor while minimizing the risk of vascular lesions (11). Moreover, it has been proven that Doppler and color Doppler ultrasonography are useful to minimize the risk of vascular damage, thus preventing postoperative complications and neurological impairments (**Figure 1**).

Brain tumors are often characterized by a hyperechoic zone of perilesional edema, which may be mistaken for tumoral infiltration. As reported, brain edema is usually less hyperechoic than tumoral infiltration; if this zone is separated from the tumor by a hypoechoic zone, then this is more likely to be edema than tumor residual (32). Nevertheless, sometimes, it may be difficult to differentiate brain edema from tumoral infiltration; in our experience, we relied on CEUS to distinguish tumoral infiltration from brain edema and on the integration of other advanced ultrasonographic modalities in order to overcome the limitations of each of them.

CEUS was performed in all patients. UCA was injected after a first B-mode ultrasonographic evaluation. Then, a 2.4-ml standard bolus of sulfur exafluoride was injected in the superior vena cava *via* central venous line, followed by a 10-ml standard bolus of saline solution.

As is already known from the literature, CEUS is a reliable solution to detect and characterize focal lesions; it is capable of highlighting tumor parenchyma and tumor–brain interface with great accuracy and distinguishing among multiple kind of lesions, thanks to the different perfusion patterns, specific for each tumor (13). In this regard, a detailed semantic pro forma for the ultrasonographic characteristics of high-grade gliomas correlating their histological characteristics has been recently proposed. According to this, grade IV tumors should appear easily discernable, with irregular crenated finger-like margins; necrosis is frequent and presented as a single large confluent area. Grade III is discernable with more difficulty and characterized by regular margins; rarely necrosis is associated and, when present, is equally likely to be multiple small foci. In our study, only grade IV glioma were evaluated due to the limitation of the sample size, and they showed ultrasonographic characteristics similar to that described in the literature (32).

**Figure 2:**
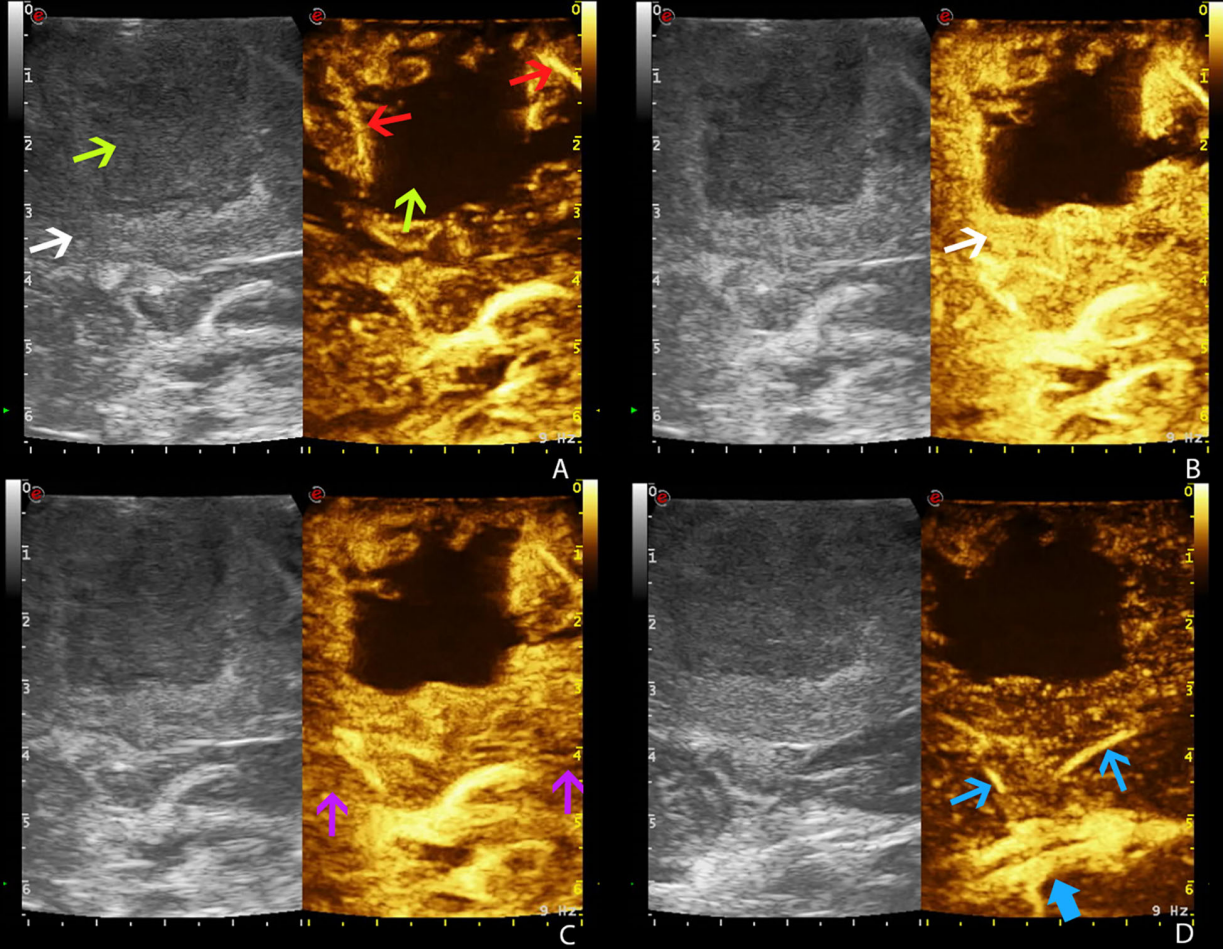
Time frame of GBM visualization by CEUS (case no. 3) (dual frame visualization: B-mode imaging on the left, contrast-enhanced ultrasound imaging on the right). Gas-filled microbubble contrast agent allows to visualize the different phases of intravascular contrast-enhancement thus visualizing the tumor angioarchitecture. **(A)** Arterial phase. **(B)** CE peak. **(C)** Parenchymal phase. **(D)** Venous phase. White arrow: GBM. Green arrows: necrotic core of GBM. Red arrows: GBM arterial supply. Purple arrows: normal brain parenchyma. Blue arrow: GBM venous drainage (blue thick arrow: internal cerebral veins).

The UCA allowed to visualize the main phases of CE in the different tumors. According to previous studies, GBM demonstrated a brief CE, characterized by rapid arterial phase (which lasted about 2–3 s before the peak of CE, which is characterized by a strong contrast enhancement of tumor tissue after UCA arrival and before the perfusion of normal brain parenchyma) (13). For the most part, the arterial feeders were clearly visible. The parenchymal phase had a typical heterogeneous and irregular pattern after UCA, characterized by nodular areas and necrotic/cystic areas, and the rapid venous phase (5–10 s) (**Figure 2**). Meningiomas showed a strong and rapid CE, with a homogeneous and strong parenchymal phase; tumor borders were clear. CEUS helped to identify the vascular rearrangement resulting from the tumor removal and to distinguish the residual tumor volume after resection. Such ultrasonographic information led us to suppose the nature of the lesion, which was later confirmed by the following pathological diagnosis.

**Figure 3:**
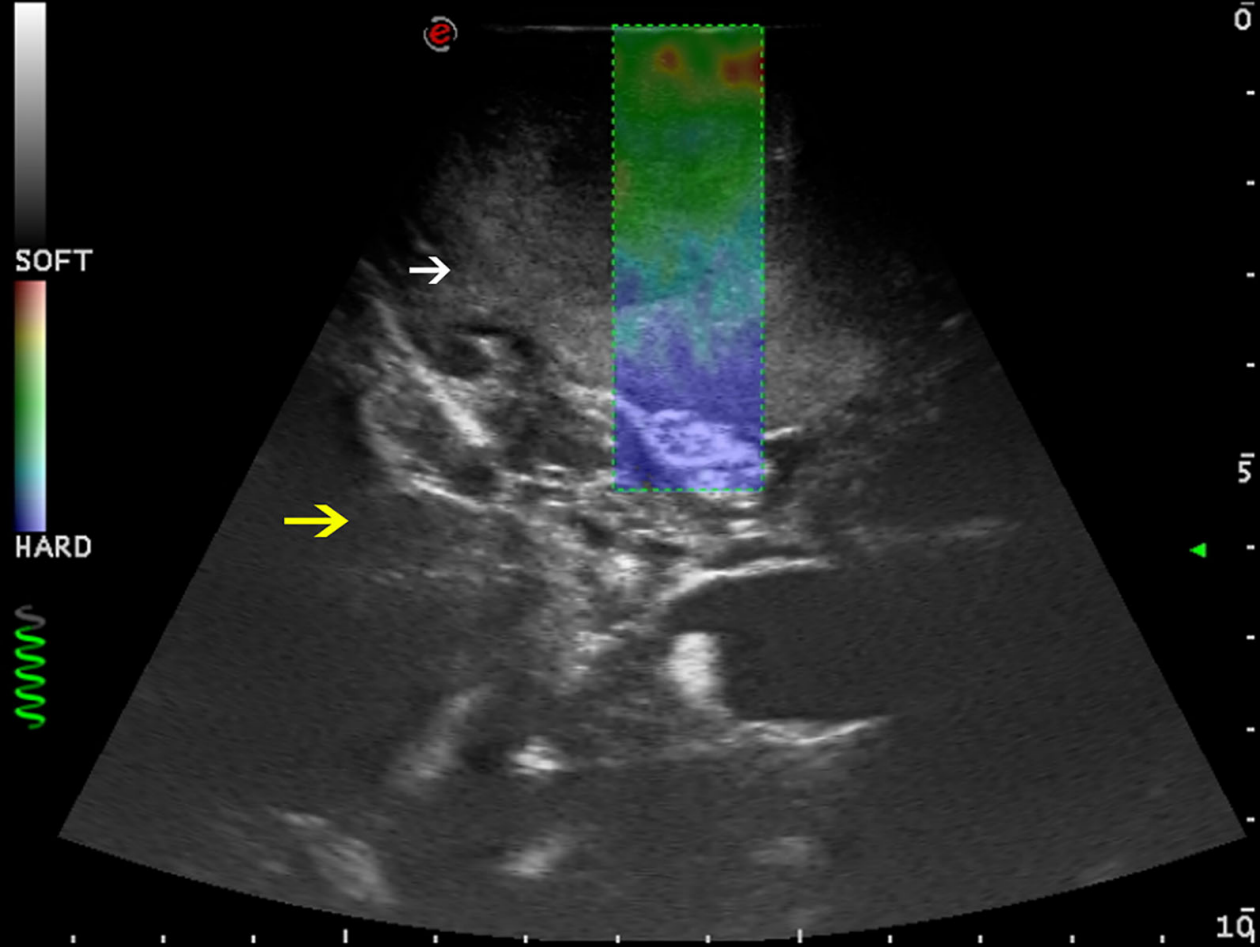
Strain elastosonography frame in a case of right parietal GBM (case no. 3). Elastosonography gives information about the stiffness of the tissue, and it is revealed through a chromatic scale. Red spots are representative of softer zones (necrotic areas); blue spots are representative of harder zones (brain–tumor interface and brain parenchyma). The core of the lesions appears to be softer than surrounding normal brain parenchyma. White arrow: GBM. Yellow arrow: surrounding normal brain parenchyma.

In those cases where elastosonography was employed, this was helpful in distinguishing between tumor and brain parenchyma and in identifying the tumor borders; our experience confirmed data from the literature (5, 16), as tumor stiffness was generally higher than the normal brain parenchyma (**Figure 3**).

Over the last few years, navigated IOUS has been developed in order to enable the fusion between intraoperative imaging (IOUS) and preoperative imaging (MRI) used for neuronavigation in order to obtain a real-time anatomical and functional feedback from IOUS imaging without interrupting surgery. Moreover, navigated IOUS promotes familiarity with IOUS for neurosurgeons, and it helps to adapt the surgical strategy on real intraoperative scenarios (2–4, 33–35).

Unfortunately, navigated IOUS is not available in all neurosurgical operating rooms. For this reason, our institution conceived a co-planar indirect navigation of IOUS, a technique based on the co-planar and coupled use of both unnavigated IOUS and standard optical neuronavigation, which had already been presented and discussed (23).

In all GBM patients, we combined IOUS and 5-ALA (36, 37). These two techniques integrate well, as they offer different perspectives of the same surgical field (5, 14, 15, 38, 39): after craniotomy and durotomy, tumor was first evaluated by IOUS and CEUS. After a proper visualization, at the earliest stages, tumor removal was conducted by the guidance of 5-ALA fluorescence. After the macroscopical completion of resection, IOUS and CEUS were adopted to see through eventually hidden residual tumor. Through this second check, we had the opportunity to spot neoplastic residual tumor tissue and to modify the surgical strategy in order to maximize the EOR.

### Representative Case: Integrated Ultrasonographic Modalities During IOUS

On April 2021, a 53-year-old woman was admitted to our institution for a right fronto-temporal meningioma. During surgery, after craniotomy, we performed a first evaluation of the lesion with B-mode in order to identify tumor borders and their characteristics. In B-mode, the lesion was hyperechogenic in relation to normal brain parenchyma, and the margins were linear and easily identified. IOUS was performed with the aid of indirect co-planar navigation, whose technique was already described (23).

After durotomy, first phenomena of brain shift and brain deformation occurred, so we performed another evaluation through B-mode, Doppler, and color Doppler; these techniques gave us the opportunity to study the vascular supply of the tumor in order to define the following surgical steps. Elastosonography was performed to evaluate *a priori* the hardness to enucleate the lesion according to its stiffness, which was higher compared to the surrounding brain parenchyma.

The perfusion pattern was then evaluated through CEUS; after injection of UCA, a 2.4-ml standard bolus of sulfur exafluoride, we observed a strong and rapid CE; the arterial phase was intense and rapid, the parenchymal phase was characterized by a homogeneous enhancement, and the venous phase was slow. These characteristics were typical of a meningioma; this suspicious was then confirmed by the pathological analysis.

During tumor resection, IOUS was used to evaluate the residual tumors and the anatomical landmarks. At the end of the resection, ultrasonography was again performed to confirm the complete removal of the tumor.

### Representative Case: Preoperative IOUS Through Iatrogenic Skull Defect

In December 2020, a comatose 50-year-old man was urgently admitted to our institution due to endocranial hypertension caused by multiples brain meningiomas. As first therapeutic intervention, decompressive craniectomy was performed. Given the good clinical postoperative outcome, he was then evaluated for a second intervention for the resection of the biggest right temporo-parietal meningioma. We performed several imaging studies through brain CT, contrasted brain MRI, and MRI angiography tractography. Due to the previous iatrogenic skull defect, also a preoperative brain US evaluation was performed. Multiple US techniques were used to plan the surgery, which were performed on February 2021. During surgery, the patient underwent IOUS, and the preoperative US images were compared to the IOUS. The precise matching between preoperative US and IOUS demonstrated the reliability of preoperative US for the surgical planning, given a proper acoustic window such as a former skull defect in selected cases. The patient had a good clinical outcome and was dismissed on the seventh postoperative day.

**Figure 4:**
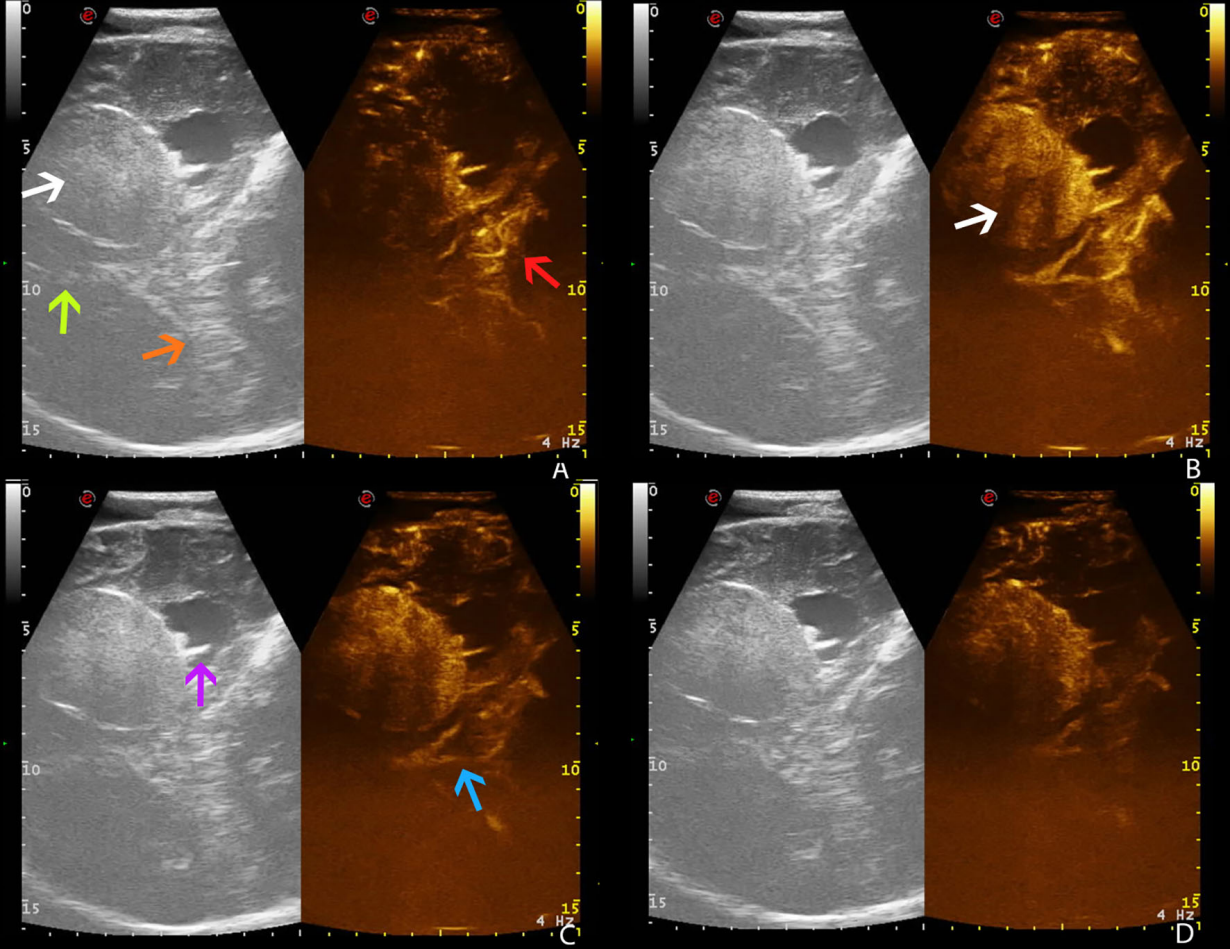
Preoperative time frame of different CEUS phases in a right temporo-parietal fibrous meningioma (case no. 5, Pre-op), using a former iatrogenic skull defect as an optimal acoustic window. **(A)** Arterial phase. **(B)** CE peak. **(C)** Parenchymal phase. **(D)** Venous phase. White arrow: fibrous meningioma. Green arrow: falx cerebri. Orange arrow: tentorium cerebelli. Red arrow: arterial supply. Purple arrow: choroidal plexus of right lateral ventricle. Blue arrow: deep venous drainage.

**Figure 5:**
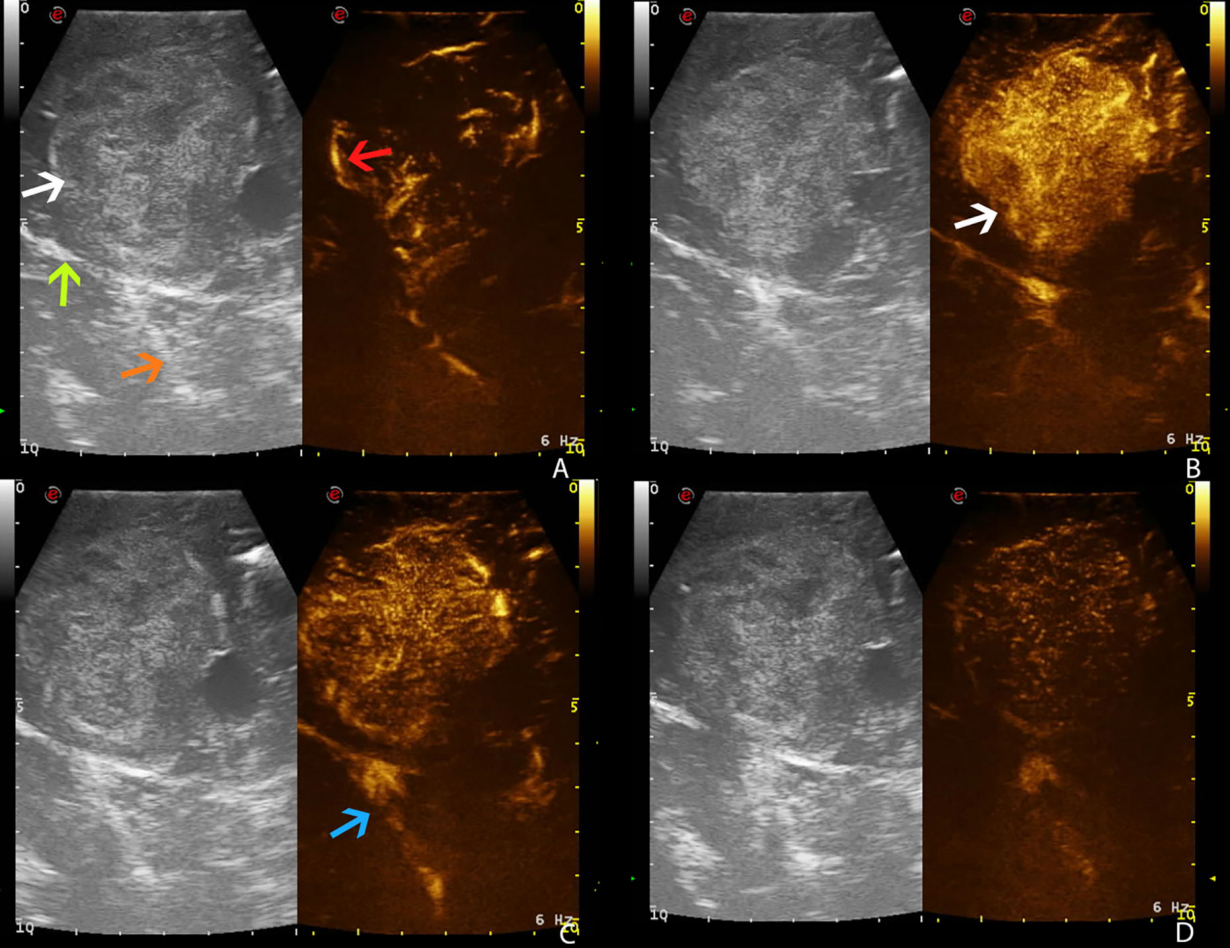
Intraoperative time frame of different CEUS phases in a right temporo-parietal fibrous meningioma (case no. 5, Intra-op), showing the exact correspondence between preoperative and intraoperative US images, thus confirming the reliability of preoperative IOUS (see for preoperative images). **(A)** Arterial phase. **(B)** CE peak. **(C)** Parenchymal phase. **(D)** Venous phase. White arrow: fibrous meningioma. Green arrow: falx cerebri. Orange arrow: tentorium cerebelli. Red arrow: arterial supply. Blue arrow: deep venous drainage.

The use of ultrasound in neurosurgery is limited because US beam is attenuated by the skull bone. In our experience, we overcame this limitation by using a previous iatrogenic skull defect due to decompressive craniectomy, which represented an optimal acoustic window. This condition revealed the skull defect as a safe and reliable acoustic window for the anatomical, vascular, and functional preoperative study, as it gave the opportunity to predict anatomical and vascular conditions that were later confirmed during surgery. This information, obtained in advance, ensures the surgeons’ deeper knowledge of the given case and allows them to plan a more accurate procedure (**Figures 4**, **5**).

### Study Limits

This preliminary study suffers from some limitations. The patient sample is small, and it lacks a control group. Despite these limitations, data from the present study equalize to data in the literature and confirm the role of IOUS in maximizing the EOR, which is strictly associated with improved postoperative outcome and patient quality of life. It is therefore possible to promote the use of IOUS as a routine practice in managing brain tumors.

## Conclusions

IOUS is becoming widely used in neurosurgery, especially in neuro-oncological surgery. It presents several advantages, as it is reliable and easy to use, non-invasive, and inexpensive. In our experience, IOUS represents a valuable tool in the intraoperative management of brain tumors.

The main point of value of IOUS consists in obtaining real-time images that can be repeated as many times as necessary, which allows the monitoring of the EOR and the intraoperative changes occurring during the surgical procedures. Moreover, IOUS is accurate in the evaluation of peritumoral microvascularization, which allows a safer and larger tumor resection while preserving normal brain parenchyma. Therefore, IOUS greatly contributes to GTR, which represents the best treatment of brain tumors.

## Data Availability Statement

The original contributions presented in the study are included in the article/supplementary material. Further inquiries can be directed to the corresponding author.

## Ethics Statement

Ethical review and approval was not required for the study on human participants in accordance with the local legislation and institutional requirements. The patients/participants provided their written informed consent to participate in this study.

## Author Contributions

Conceptualization, GG, SM, and RM. Methodology, GG. Investigation, SM. Resources, LB, RC, FP, GSa and RD. Data curation, SM. Writing—original draft preparation, SM. Writing—review and editing, GG, PP, and GSc. Visualization, ST, GU, MM, MP, and RG. Supervision, DI and RM. Project administration, DI. All authors contributed to the article and approved the submitted version.

## Conflict of Interest

The authors declare that the research was conducted in the absence of any commercial or financial relationships that could be construed as a potential conflict of interest.

## Publisher’s Note

All claims expressed in this article are solely those of the authors and do not necessarily represent those of their affiliated organizations, or those of the publisher, the editors and the reviewers. Any product that may be evaluated in this article, or claim that may be made by its manufacturer, is not guaranteed or endorsed by the publisher.
